# The etiological effect of a new low-frequency *ESR1* variant on Mild Cognitive Impairment and Alzheimer’s Disease: a population-based study

**DOI:** 10.18632/aging.101548

**Published:** 2018-09-16

**Authors:** Xiaoling Li, Xiaoquan Zhu, Wandong Zhang, Fan Yang, Juan Hui, Jiping Tan, Haiqun Xie, Dantao Peng, Lihua Ma, Lianqi Cui, Shouzi Zhang, Zeping Lv, Liang Sun, Huiping Yuan, Qi Zhou, Luning Wang, Shige Qi, Zhihui Wang, Caiyou Hu, Ze Yang

**Affiliations:** 1 Graduate School of Chinese Academy of Medical Science and Peking Union Medical College, Beijing 100001, P.R.China; 2 The MOH Key Laboratory of Geriatrics, Beijing Hospital, National Center of Gerontology, Beijing 100730, P.R.China; 3 Department of Pathology and Laboratory of Medicine, Faculty of Medicine, University of Ottawa, Ottawa K1H 8M5, Canada; 4 Human Health Therapeutics, National Research Council of Canada, Ottawa K1A 0R6, Canada; 5 Department of Geriatric Neurology, Chinese PLA General Hospital, Beijing 100730, P.R.China; 6 Department of Neurology, Affiliated Foshan Hospital of Sun Yat-sen University, Foshan, 528000, P.R.China; 7 China-Japan Friendship Hospital, Beijing 100029, P.R.China; 8 253 Hospital of PLA, Huhehot 010051, P.R.China; 9 Department of Neurology, 401 Hospital of PLA, Qingdao, Shandong 266100, P.R.China; 10 Department of Neurology of Beijing Geriatric Hospital, Beijing 100095, P.R.China; 11 National Rehabilitation Aids Research Center, Ministry of Civil Affairs, Beijing 100176, P.R.China; 12 National Center for Chronic and Non-communicable Diseae Control and Prevention, Chinease CDC, Beijing 100050, P.R.China; 13 Department of Neurology, Jiangbin Hospital, Guangxi Zhuang Autonomous Region, Nanning 530021, P.R.China

**Keywords:** AD, MCI, *ESR1*, cholesterol, Aβ

## Abstract

Latent genetic variations of cholesterol metabolism-related genes in late-onset Alzheimer’s disease, especially, as well as in mild cognitive impairment pathogenesis are still to be studied extensively. Thus, we performed the targeted-sequencing of 12 nuclear receptor genes plus *APOE* which were involved in cholesterol content modulation to screen susceptible genetic variants and focused on a new risk variant *ESR1* rs9340803 at 6q25.1 for both late-onset Alzheimer’s disease (OR=3.30[1.84~4.22], *p*<0.001) and mild cognitive impairment (OR=3.08[1.75~3.89], *p*<0.001). This low-frequency variant was validated in three independent cohorts totaling 854 late-onset Alzheimer’s disease cases, 1059 mild cognitive impairment cases and 1254 controls from nine provinces of China mainland. Preliminary functional study on it revealed decreased *ESR1* expression in vitro. Besides, we detected higher serum Aβ1-40 concentration in participants carrying this variant (*p*=0.038) and lower plasma total cholesterol level in this variant carriers with late-onset Alzheimer’s disease (*p*=0.009). In summary, we identified a susceptible variant which might contribute to developing mild cognitive impairment at earlier stage and Alzheimer’s Disease later. Our study would provide new insight into the disease causation of late-onset Alzheimer’s disease and could be exploited therapeutically.

## Introduction

Late-onset Alzheimer’s disease (LOAD, OMIM: 104310) is a complex neurodegenerative disease with polygenic background. It’s characterized by extracellular senile plaques (SPs) of which the core protein is β-Amyloid peptide (Aβ), mostly 40- or 42-amino acid peptides, and intracellular neurofibrillary tangles (NFTs) in the brain [1]. A variety of genetic as well as environmental factors have long been believed to associate with LOAD, with *APOE* as the strongest genetic factor and ageing as the most influential risk factor. Though a number of distinct loci and risk genes have been discovered by several genome-wide association studies (GWAS), the genetic etiology of LOAD remains largely elusive [2–4].

Multiple studies have demonstrated the correlation between altered cholesterol levels and increased Aβ formation in cellular and animals models of LOAD [5–7]. Specifically, optimal cholesterol content in neurons is reported to be critical to the stability of the brain microenvironment [8]. In this respect, several cholesterol metabolism-related nuclear receptor (NR) molecules in LOAD brain have been reported by independent studies [9–11]. However, whether possible NR gene variations, which may functionally cause disturbed cholesterol content and impair normal cholesterol activity, would promote Aβ formation and thus induce LOAD still warrants to be studied extensively. What’s more, exploration on the effect of AD-related NR gene variations in Mild Cognitive Impairment (MCI) patients is still lacking. In this context, we speculate that latent variations of NR genes which would exacerbate Aβ-induced memory impairment through acting on cholesterol modulation are likely to participate in disease development of AD continuum including MCI due to AD as the prodromal phase.

Therefore, the present study is designed to identify disease risk-linked genetic variations of NR genes and explore the possible pathogenic mechanisms basing on the relative large case-control sample groups and with use of bioinformatic tools. With all these efforts, we intend to provide some new knowledge about the effect of potential genetic variations on cholesterol content subtlety as well as consequent Aβ production, which might have impact on MCI and AD incidence.

## RESULTS

### Identification and replication of AD- related variants

**Figure 1:**
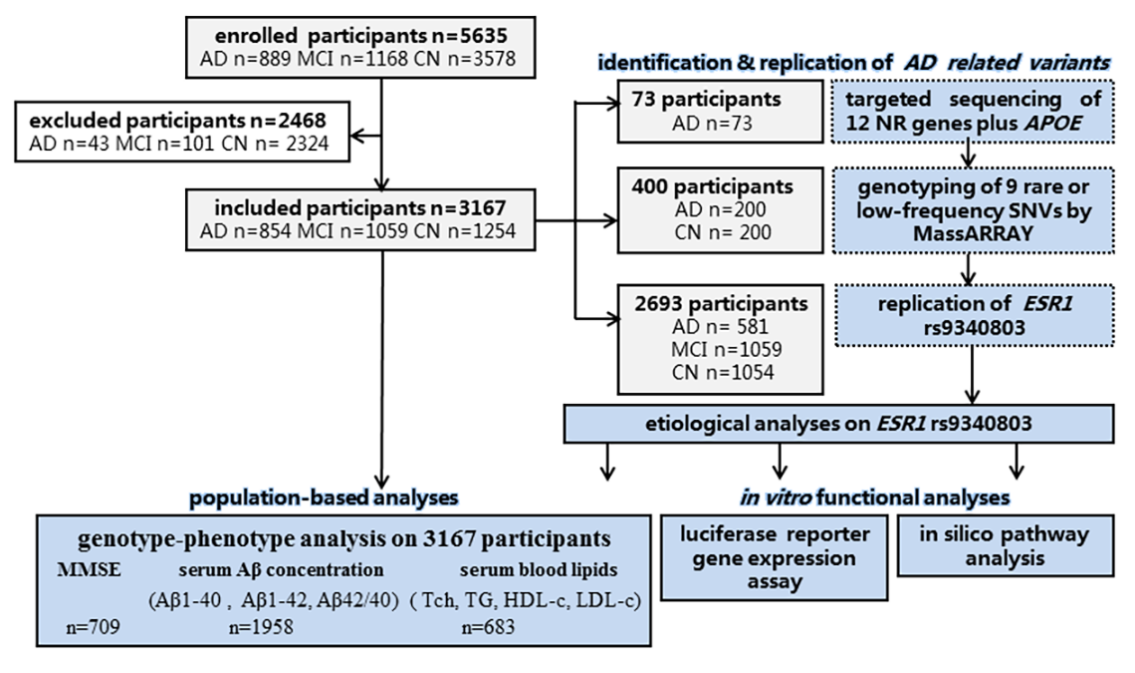
Flow diagram of the present study. n: number; CN: cognitively normal control.

**Figure 2:**
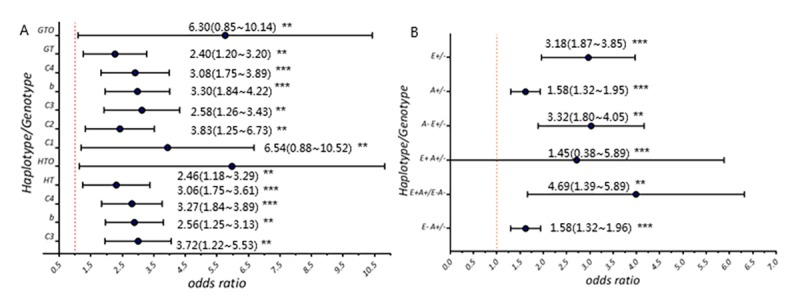
**Risk of *ESR1* rs9340803 in AD and MCI cases.** (**A**) Allele frequency and genotype frequency of *ESR1* rs9340803. C1: cohort 1 (200 AD case vs. 200 controls); C2: cohort 2 (580 AD cases vs. 1054 controls); C3: combined cohort (854 AD cases vs. 1254 controls); C4: CI cases vs. controls; HT: haplotype; HOT: haplotype in individuals aged 70 and more; GT: genotype; GOT: genotype in individuals aged 70 and more. (**B**) Comparisons between CI and CN individuals on rs9340803 minor allele distribution. E+: *ESR1* rs9340803 G allele carrier; E-: *ESR1* rs9340803 A allele carrier; A+: *APOE* ε4 carrier; A-: non-*APOE* ε4 carrier.

After targeted sequencing of 12 NR genes plus *APOE* which were involved in cholesterol metabolism modulation on 73 LOAD cases first, 9 out of 1690 rare or low-frequency SNVs enriched in AD samples were paid on great interest (Table 1; Supplementary Tables S2, S3). Genotyping of these candidate variants on 200 LOAD cases and 200 controls subsequently revealed that rs9340803 A>G in *ESR1* (ENSG00000091831) intron 4 at 6q25.1 (MAF<1%) was associated with AD risk. Replication of this *ESR1* variant in three independent sample groups totaling 854 AD cases, 1059 MCI cases and 1254 controls affirmed that this variant was risk-associated for both AD and MCI (Fig. 1, Fig. 2). Additionally, genetic drift was ruled out because this SNP was of low-frequency (MAF< 2%) in all subpopulations by referring to the 1000 Genomes Project. Baseline characteristics of participants in the present study referred to Supplementary Table S1.

**Table 1:** Candidate SNVs for genotyping.

SNV	Position	GENE	MAF in 1000G	MAF in 73 LOAD
Chr12 g.48272978 C>A	chr12:48272978	*VDR*	-	6/73
rs9658164 G>A	chr6:35392709	*PPARD*	0.005	6/73
rs138110733 G>A	chr9:137265442	*RXRA*	0.002	4/73
rs9340803 A>G	chr6:152163967	*ESR1*	0.004	5/73
Chr19 g.45406107 A>G	chr19:45406107	*ApoE*	-	8/73
rs116932128 G>A	chr17:38246314	*THRA*	0.018	12/73
rs139374285 C>T	chrX:66914636	*AR*	0.008	5/73
rs78087244 C>T	chr6:152449830	*ESR1*	0.041	7/73
rs9397459 G>A	chr6:152265659	*ESR1*	0.05	7/73

-: no MAF information in database.

### Population-based etiology analysis of AD-related *ESR1* rs9340803

### *ESR1 variant distribution among AD/MCI/CN*

As shown in Fig. 2, this *ESR1* variant was demonstrated to associate with AD risk by comparisons between AD cases and controls from our large sample collection and revealed a 3.30-fold risk of developing AD (Fig.2A; Table S3). Higher percentage of the *ESR1* rs9340803 minor allele was shown in both AD and MCI cases as compared to that in CNs (4.53%, 4.23% vs. 1.37%; *p*<0.001), whereas the frequency didn’t differ significantly between AD and MCI cases (*p*=0.059). Further analyses stratified by sex and age group showed same trend existing as more minor allele carriage in AD and MCI cases (Table S4). Besides, we took *APOE* into consideration for further stratification, which turned out more *APOE*ε4- AD cases (4.19%) and more *APOE*ε4+ MCI cases (3.37%) with this *ESR1* variant separately.

When comparing MCI cases to CNs solely, the disease risk was elevated by 2.08-fold with this *ESR1* variant (Fig. 2A b; Table S3). There were more MCI cases with A/G heterogeneous genotype of this *ESR1* variant than controls with that genotype when stratified as sex and age group (*p*<0.01; *p*<0.01). MCI cases carrying the *ESR1* variant but without *APOE*ε4 also displayed high disease risk relative to their CN counterparts (OR: 3.24 (1.74~4.20), *p*<0.001). However, to carriers with both the *ESR1* variant and *APOE* ε4, there was no significant difference (*p*=0.190).

### *ESR1 variant distribution among CI/CN*

AD and MCI patients were then combined together as CI cases who had amnesic memory problem in our study. As a result, these cases appeared to possess higher frequency of *ESR1* rs9340803 heterozygous genotype (4.36% vs. 1.37%, *p*<0.001) comparing with controls, indicating the minor allele of rs9340803, like *APOE*ε4, as a risk factor, which was manifested by a 3.18-fold of risk in the occurrence of memory problem in CI cases (Fig. 2B; Table S5). The independent effect of the G allele of this SNP apart from *APOE*ε4 was somewhat greater than that of *APOE*ε4 (OR= 3.23 (1.80~4.05) vs. 1.58 (1.32~1.90); Table S5). Logistic regression analysis with age, sex and *APOE* fixed confirmed it (Table S6). While, the joint action of the G allele and *APOE*ε4 greatly increased the cognitive anomaly risk, with relative to individuals with both rs9340803 major allele homozygosity and non-*APOE*ε4 (OR= 4.69 (1.39~5.89), *p*=0.006). Besides, the frequency of the minor allele of this SNP also differed between CI and CN subgroup members as categorized by age and sex separately, with more old and male cases with the minor allele (Table S7). Specifically, among CI cases without *APOE*ε4, this *ESR1* variant impacted the risk of disease incidence for not only females but also males (OR=2.74, *p*=0.048; OR=3.52, *p*=0.002; Table S8). Gene-gene and gene-environment interaction analyses were additionally done which further validate the joint effect of this *ESR1* variant, *APOE*ε4 and aging, with high-risk haplotype G-T-C occupying the potential of increasing disease risk to 2.46 (1.18~3.29)-fold for the elderly all and to 6.54 (0.88~10.52) if aged 70 and older (Supplementary Table S9).

### *AD-associated traits attributed to ESR1 rs9340803*

*MMSE of AD and MCI cases.* The median MMSE aggregated score (median (Q)) of AD cases was 15(9, 21). There were no statistically significant differences of MMSE scored by AD cases with and without this *ESR1* variant (*p*=0.874), with and without *APOE*ε4 (*p*=0.178), or between male and female cases (*p*=0.103), respectively (Table S10). While divergence reached the statistical significance between older (14(9, 20)) and younger (18(13, 22)) elderly (*p*<0.001). Further *APOE*ε4-stratified analysis still showed no divergences comparing carriers of this *ESR1* variation with non-carriers (*p*=0.423; *p*=0.710).

The median MMSE aggregated score (median (Q)) of MCI cases was 15(11, 23). There were no statistically significant differences of MMSE scored by MCI cases with and without this *ESR1* variant (*p*=0.488), with and without *APOE*ε4 (*p*=0.078), respectively, but between male (17(13, 21)) and female (14(11, 17)) cases(*p*<0.001). While divergence reached the statistical significance between older (15(11, 18)) and younger (16(13, 19)) elderly (*p*=0.006). Further *APOE*ε4-stratified analysis still showed no divergences comparing carriers of this *ESR1* variation with non-carriers (*p*=0.710).

The median MMSE aggregated score (median (Q)) of CI cases was 15(11, 19). There were no statistically significant differences of MMSE scored by CI cases with and without this *ESR1* variant (*p*=0.504), with and without *APOE*ε4 (*p*=0.986), respectively, but between male (17(13, 21)) and female (14(11, 17)) cases(*p*<0.001). While divergence reached the statistical significance between older (15(11, 18)) and younger (16(13, 19)) elderly (*p*<0.001). Further *APOE*ε4-stratified analysis still showed no divergences comparing carriers of this *ESR1* variation with non-carriers (*p*=0.806).

*Serum Aβ-oligomer concentrations.* For the overall individuals, serum Aβ1-40, Aβ1-42 concentration and Aβ1-42/1-40 were observed to differ significantly among three subgroups (*p*<0.001, *p*<0.001, *p*<0.001). To be specific, Aβ1-40 concentration and Aβ1-42/1-40 were different in subjects from three subgroups but Aβ1-42 concentration didn’t differ between MCI cases and CNs (*p*=0.052; Table S11). When comparing CI cases to controls subsequently, same trend persisted (*p*<0.001, *p*<0.001, *p*<0.001) (Table 2). Thus, Aβ1-40, Aβ1-42 and Aβ1-42/1-40 were indicated to potentially constitute the serum Aβ profile along AD disease continuum in our study. Whereas, only divergence of serum Aβ1-40 concentration reached the statistically significant difference for E+/- (*p*=0.038) subclass with higher Aβ1-40 (47.12(31.46, 53.82) pmol/L) concentration in participants carrying this *ESR1* variant, which might imply Aβ1-40 as a more stable biomarker in the blood. Besides, Aβ1-40 concentrations in participants with *APOE*ε4 genotype showed a lack of statistically significant difference in comparison with those with non-*APOE*ε4 (p=0.010; Table S12).

**Table 2:** Analyses on serum Aβ-oligomer concentrations (pmol/L).

	AD	MCI	CN	*p*	CI	CN	*p*
Aβ40	39.69 (21.98, 53.50)	29.63 (15.47, 47.92)	16.52 (4.84, 44.64)	<0.001	32.67 (16.52, 49.81)	16.52 (4.84, 44.64)	<0.001
Aβ42	3.68 (2.17, 5.72)	2.74 (1.22, 4.60)	2.25 (1.08, 4.15)	<0.001	3.06 (1.41, 4.87)	2.25 (1.08, 4.15)	<0.001
Aβ42/40	0.103 (0.078, 0.135)	0.097 (0.073, 0.117)	0.130 (0.081, 0.254)	<0.001	0.10 (0.07, 0.12)	0.13 (0.08, 0.25)	<0.001
	E+	E-		*p*	A+	A-	*p*
Aβ40	35.56 (14.84, 57.44)	30.14 (11.98, 48.21)		0.038	33.05 (15.25, 52.09)	29.69 (11.48, 47.80)	0.01
Aβ42	3.76 (1.43, 5.82)	2.80 (1.28, 4.67)		0.124	2.88 (1.33, 4.91)	2.80 (1.29, 4.63)	0.468
Aβ42/40	0.098 (0.080, 0.125)	0.102 (0.075, 0.138)		0.563	0.099 (0.073, 0.124)	0.103 (0.076, 0.141)	0.026

*Plasma cholesterol levels.* As the notion that elevated plasma cholesterol is a risk factor for AD, four representative indexes of Plasma cholesterols (TC, TG, HDL-c and LDL-c) were tested. In our study, TC (*p*<0.001) and HDL-c (*p*=0.050) levels were each significantly differed among individuals from three subgroups (Table S13). As regards to further comparison between CIs and CNs, there were statistically significant divergences as for TC (*p*<0.001) and TG concentrations (*p*=0.022), respectively. While, only lower TC levels were observed in the *ESR1* variant carriers as compared to those non-carriers (*p*=0.045; Table S14). These results didn’t change when CI cases (*p*=0.04) and AD cases only (*p*=0.009) carrying the *ESR1* variant were considered (Table 3). However, there were no statistically significant difference for TC levels in MCI cases carrying the *ESR1* variant with relative to the counterparts (*p*=0.494). Additionally, statistically significant difference was not either showed for TC level between *APOE*ε4 carriers and non-ε4 carriers (*p=*0.294).

**Table 3:** Analyses on blood lipids levels (mmol/L).

	All			CI			AD		
	E+	E-	*p*	E+	E-	*p*	E+	E-	*p*
Tch	3.96 (1.39, 5.42)	4.79 (3.96, 5.48)	0.045	3.73 (1.30, 5.04)	4.61 (3.77, 5.37)	0.04	3.96 (1.39, 5.42)	4.79 (3.96, 5.48)	0.009
TG	1.58 (0.92, 4.33)	1.28 (0.90, 1.92)	0.315	1.45 (0.89, 4.39)	1.35 (0.90, 2.00)	0.505	1.58 (0.92, 4.33)	1.28 (0.90, 1.92)	0.176
HDL	1.35 (1.11, 1.57)	1.29 (1.10, 1.54)	0.729	1.34 (1.10, 1.58)	1.29 (1.10, 1.51)	0.669	1.35 (1.11, 1.57)	1.29 (1.10, 1.54)	0.38
LDL	2.47 (1.69, 3.52)	2.76 (2.23, 3.31)	0.293	2.44 (1.62, 3.24)	2.74 (2.19, 3.30)	0.159	2.47 (1.69, 3.52)	2.76 (2.23, 3.31)	0.066

*Relation of ESR1 rs9340803 with plasma cholesterol levels, serum Aβ oligomer concentrations and MMSE.* Serum Aβ1-40 and Aβ1-42 concentrations separately correlated to MMSE score in our study samples (*p*<0.001, *p*<0.001). Correlation of TC level to MMSE score also existed in CI cases (*p*<0.001). What’s more, partial correlation analyses turned out that TC (*p*<0.001), TG (*p*<0.001) and LDL-c (*p*<0.001) correlated to Aβ1-40 in concentration, respectively. Positive correlations between the TC level and Aβ oligomers were noted in individuals the *ESR1* variant (*p*<0.001) even when stratified by *APOE*ε4 (*p*<0.001).

### Functional study of AD-related *ESR1* rs9340803

### *ESR1 rs9340803 G variant potentially damages ESR1 transcription*

This intronic SNP residing 45bp downstream of exon4 was predicted by the *in silico* prediction programs to broke the binding motif for a splice auxiliary protein hnRNP H1 and generate a hnRNP A1 binding motif which would promote exon 4 skipping, impairing the normal regulation activity of intronic splicing process of *ESR1* pre-mRNA, which might down-regulate the transcription of *ESR1* (Fig. S1).

### *ESR1 rs9340803 variation affects the expression of the gene*

**Figure 3:**
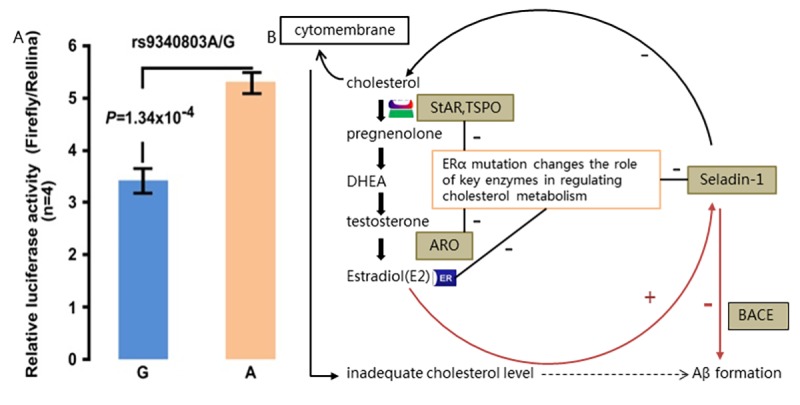
**Functional study of rs9340803.** (**A**) Relative luciferase activity assay performed with 293T cell line (repeated four times). Firefly luciferase expression was normalized using activity of renilla luciferase. Ratio of the normalized firefly luciferase expression to that of control was used to represent relative luciferase activity. Data represented the mean+s.d. (**B**) Postulated pathway diagram of estradiol-ER-cholesterol-Aβ formation cycle. Cholesterol-originated estrogen binds to ERα and regulates key enzymes in the cholesterol metabolism. Decreased *ESR1* expression caused by rs9340803 A to G variation may disrupt the balance of adequate cholesterol content in the cytoplasm and on the cytomembrane of neuron as a result of decreased ERα activity on those key enzymes, promoting more Aβ production. Red line indicates the direct effect of estrogen ligand and Erα through BACE and black line refers to indirect effect by rugulation of cholesterol content. DHEA: dehydroepiandrosterone; StAR: steroidogenic acute regulatory protein; TSPO: translocator protein; ARO: aromatase.

In accordance with previous prediction, dual-luciferase reporters assay showed statistically significant decreased luciferase expression (*p*<0.001; Fig 3A). This result indicated decreased expression of *ESR1* transfected with homozygous G allele, conferring the potential functional relevance of this *ESR1* A>G variant.

### *In silico pathway analysis of Estrogen-ERα-cholesterol in brain*

Estrogen deficiency and altered lipid profile are considered significant risk factors for AD. Cholesterol is the substrate for estrogen synthesis and the possible interactions between cholesterol and estrogens in the etiology of AD, may be influenced by the cholesterol metabolism [12]. Bioinformatic pathway analysis regarding the estrogen-ERα-cholesterol cycle was thus done on the basis of *ESR1* gene function, previous study results and our preliminary results (Fig. 3). Estradiol (E2 as the most form of estrogen) originating from cholesterol by source, was recognized to bind with estrogen receptor (ERα mostly in hippocampus) at first and promote complex biological outcomes in brain regions at last, playing a protective role in neurodegenerative diseases. Cholesterol content in the cytoplasm and on the membrane was modulated in some way by key enzymes, which can be regulated by interaction of factors like estrogen response elements (EREs) with ERα directly or by estrogen after bind with activated Erα (Fig. 3B).

## DISCUSSION

We identified the association of a new *ERS1* variant with both AD and MCI risk (AD: OR=3.30(1.84~4.22), *p*<0.001; MCI: OR=3.08(1.75~3.89), *p*<0.001) basing on a large cohort (854 AD cases, 1059 MCI cases, 1254 controls). It’s important as we show, for the first time, that this variant with AD risk is also MCI-associated. Meanwhile, our results are derived from population-based study with samples from multiple regions across China mainland relative to similar studies [13–15]. Our findings indicate that low-frequency susceptibility alleles would contribute to the risk of developing both MCI and AD and offer insight into disease causation. The present study also provides supporting evidence for MCI being the specific early-stage AD. Moreover, we emphasize on the significance of this *ESR1* variant which potentially possessed etiological relation to MCI and AD, because it might have implication on preclinical intervention usage.

Notably, we also revealed serum Aβ concentrations related to AD and MCI cases with the *ESR1* variant. This AD risk-associated variant could affect by increasing the Aβ-oligomer concentrations, that is, higher Aβ1-40 concentration is observed in participants carrying this *ESR1* variant in our study (Table 2). Besides, we observed the molecular epidemic characteristics of neurotoxic Aβ isoforms: most Aβ1-40 in AD cases and less in MCI cases while lest in controls; most Aβ1-42 in AD cases while less in MCI cases and controls. That is may mainly because Aβ42 oligomers, which are more hydrophobic and prone to build up, have been reported to emerge preceding Aβ40 during the Aβ cascade process, and Aβ40 oligomers accumulate overwhelmingly as the most form of Aβ species [16,17]. Our results underlie the important role of *ESR1* genetic variation in Aβ pathology, and the sequence of Aβ isoforms at different times along the continuum of the degenerative process.

This *ESR1* variant is also found to be in correlation with altered blood lipid fractions in AD and MCI cases of our study. But significantly lower plasma TC level is only seen in *ESR1* variant carriers with AD diagnosis (Table 3), which can be partially explained that low plasma TC levels could be a result of AD pathology and linked to increased insulin resistance especially in AD patients lack of estrogen protection [18,19]. Lipid metabolism dysregulation is found systematically and in brain among patients with LOAD [7]. In fact, disturbed cholesterol content which would impair the cognitive function have been substantially explored, while, the relation between *ERS1* variants and cholesterol level in AD and MCI patients is till poorly understood [20,21]. Considering the fact that altered cholesterol levels accompanied by Aβ accumulation increasing leads to AD development [22–24], we pay much attention to the relationship among *ESR1* variation, Aβ concentration and cholesterol levels in AD and MCI cases. In our study, statistically significant correlations exist between Aβ1-40, Aβ1-42 and Aβ1-42/1-40 each and TC levels in *ERS1* variant carriers, implying a link between abnormal cholesterol content and Aβ production in brain in the context of *ESR1* variation, similar to studies on other cholesterol metabolism-related genes like *CLU* and *SORL1* [13,14]. Whereas, knowledge about the impact of *ESR1* variation on cholesterol content and Aβ production in AD patients, particularly in MCI cases, is still lacking.

To explain the possible functional mechanism of impact of *ESR1* variation on cholesterol level and Aβ in AD, even in MCI development, we suppose that along with aging, brain microenvironment of individuals carrying this *ESR1* variant are much vulnerable to specific environmental factors such as altered lipids levels, thus mutant ERα which partakes in the cholesterol metabolism, would exacerbate cholesterol disturbance and triggers a series of reaction including continually Aβ production and lead to neuronal apoptosis in brain tissues, inducing MCI early and eventual AD later. Our study implies some new ideas on MCI as early phrase of AD development. Correspondingly, bioinformatic pathway analysis ulteriorly hint us that ERα might modulate, in some way, the content of cholesterol in brain by inhibiting several key cholesterol-related enzymes which all are expressed in the hippocampus and regulate the estrogen synthesis at different steps (Fig. 3). Therefore, this variation on *ESR1*, which might have functional impact by modulating the cholesterol content in brain and thus promoting Aβ production, could be a causal factor among the complex genetic pathological basis of AD.

As one of NR family member, estrogen receptor α (Erα, P03372), encoded by the *ERS1* gene, is highly expressed in brain regions especially the hippocampus and hypothalamus which are associated with memory and cognitive performance [25–27]. ERα is activated by binding with estrogen and estrogen-ERs has been proposed to partake in cholesterol metabolism and Aβ accumulation in brains from LOAD patients [28]. In vitro gene expression assay in our study reveals decreased ESR1 expression with homozygous risk alleles. So, it is reasonable to infer that this *ESR1* variant might interfere the amount and activity of ERα, leading to loss of neuroprotective effects of estrogen with promoted Aβ production included. Bioinformatic pathway analysis ulteriorly hints us that ERα might take part in modulating, in some way, the content of cholesterol in brain by inhibiting several key cholesterol-related enzymes which all are expressed in the hippocampus and regulate the estrogen synthesis at different steps (Fig. 3), on the ground of local estrogen synthesis with cholesterol as precursor in the hippocampus of adult brain [23,28].

In addition, variants of several cholesterol metabolism modulation-related genes, nuclear receptor encoding genes for example, has been reported by a series of studies to alter the cholesterol level in brain and thus increase Aβ production, exacerbating the cognitive decline as a result [29–31,10]. Based on the overall findings, we, therefore, propose that the *ESR1* variant identified in our study might act by perturbing the subtlety of cholesterol content in brain, to promote Aβ production as well as increase Aβ toxicity, and consequently induce cognitive decline which is manifested by worsening cognitive symptoms slightly in the elderly with MCI and severely in AD seniors, whereby participating in the pathological process of AD.

What needs to be emphasized is that we conducted multiple tests based on a relative large sample size. Although Bonferroni correction was adopted in pairwise comparison, there was still a high possibility of type I error. Therefore, results of this study are mostly exploratory, and the corresponding conclusions need to be verified by subsequent studies. No doubt intensive study of the functional effect of this variant is warranted for our following research, we hope findings of the present study may aid in understanding more about the pathological underpinnings of AD.

## MATERIALS AND METHODS

### Subjects

A total of 5635 Han Chinese participants were enrolled to our study over a 5-year period (from November 2012 through December 2017) and 3167 eligible ones (57.4% were female) were investigated finally, consisting of 854 cases with LOAD (median age(Q):77.50 [14] year-old), 1059 with mild cognitive impairment (MCI; median age:73.0 [12] year-old) and 1254 age- and region-matched cognitively normal controls (CNs; median age: 65.0 [10] year-old) according to designated inclusion and exclusion criteria (Supplementary Methods). Enrollment basing on cluster random sampling among urban residences had been undertaken from the Beijing Hospital, the Jiangbin Hospital of Guangxi Zhuang Autonomous Region, the Affiliated Foshan Hospital of Sun Yat-sen University, the Chinese Center for Disease Control and Prevention (CDC) and other seven hospitals, dispersing nine provinces across northern and southern China mainland.

All of the individuals affected with dementia due to LOAD were diagnosed using the criteria of the National Institute of Neurological and Communicative Disorders and Stroke and the Alzheimer’s Disease and Related Disorders Association (NINCDS-ADRDA) [32]. Neuroimaging was used to support the probable/possible AD diagnosis based on atrophy in the medial temporal lobe, according to the Fazekas criteria by brain structural MRI [33]. The diagnoses of MCI were reached according to the core clinical criteria of the National Institute on Aging and the Alzheimer’s Association (NIA-AA) [34]. A consensus clinical diagnosis was established by at least two neurologists.

Cognitively normal control subjects were recruited from community-based and hospital-based elderly following the criteria as I) age 50 years or greater; II) no history of suggesting of brain diseases or cognitive decline; and III) no obvious hemorrhage or infarction in brain imaging.

All procedures including blood sample collection were approved by the ethical review boards at the centers involved in this study and written informed consents was obtained from each subject or proxy. The study was performed according to the principles of the Helsinki Accord.

### Identification and replication of candidate variants

### *Targeted sequencing of 12 candidate NR genes and APOE*

Twelve cholesterol metabolism-related NR genes (*VDR*, *THRA*, *ESR1, ESR2, LXRB, PPARA, PPARB, PPARG, AR, GR, RXRA* and *RXRB*) were selected for next-generation sequencing on 73 LOAD patients (Supplementary Methods). We incorporated *APOE* into the gene panel as well.

### *Candidate variants selection*

To discover new, rare or low-frequency variants that are associated with LOAD, we applied several rigorous analysis steps and selected candidate SNVs enriched in LOAD samples for subsequent association tests basing on given criteria. Detailed selection process referred to Supplementary Methods.

### *Genotyping of 9 variants using iPLEX Gold chemistry*

Candidate rare or low-frequency SNVs were genotyped on 200 LOAD cases and 200 controls using the MassARRAY Compact system (Sequenom, San Diego, CA). Quality control of genotyping was carried out afterwards.

### *Large scale population screening on ESR1 rs9340803 and APOE*

Among multiple population from nine provinces across northern and southern China mainland, additional 2694 individuals composed of 581 LOAD cases, 1059 MCI cases and 1054 controls were screened on *APOE*ε4 and *ESR1* rs9340803 genotypes.

### Population-based phenotype analyses

### *Detection of Aβ-oligomer concentrations in the serum*

On recognition that as an important biomarker of AD, Aβ can unwittingly accumulate in the brain for years, disrupting nerve connections essential for thinking and memory, and can enter systematic blood via the blood-brain barrier, we detected the concentration of serum Aβ-oligomers exploiting the Human/Rat Amyloid (40/42) ELISA Kit (WAKO; Osaka, Japan).

### *Detection of blood lipid fractions levels*

Given that several studies have demonstrated high concentrations of plasma cholesterol increased the risk of AD, fasting lipid fractions (total cholesterol [TC], triglycerides [TGs], high-density lipoprotein cholesterol [HDL-C] and low-density lipoprotein cholesterol [LDL-C]) were studied.

### Preliminary analysis on the functional effect of *ESR1* rs9340803

### *In silico damaging prediction of ESR1 rs9340803*

Splicing factor analysis and damaging prediction was conducted by exploiting several online websites, that is MutationTaster (http://www.mutationtaster.org/), Sfmap (http://sfmap.technion.ac.il/) and Human Splicing Finder (http://www.umd.be/HSF3/index.html), to explore on the potential effect of *ESR1* rs9340803 A to G variation.

### *In vitro expression assay of mutated ESR1*

Firefly luciferase and renilla luciferase reporter gene expression assay and kymographs were performed to analyze the expression of *ESR1* transfected with wild-type or variant RTN3 constructs on 293T cell line for preliminary functional exploration on *ESR1* variation.

### *Bioinformatic pathway analysis*

KEGG (http://www.genome.jp/kegg/pathway.html) and Gene Ontology (http://www.geneontology.org/) were utilized for in silico pathway analysis linking *ESR1* to AD pathogenesis.

### Statistical analysis

Genotypes were evaluated for departure from Hardy-Weinberg equilibrium (HWE) in the controls using chi-squared tests. Variants with *p*<0.05 were considered to deviate from HWE. Minor allele frequency (MAF) of variants were used as the risk allele frequencies and 4% was defined as the prevalence of AD [1].

Data were presented as number and percentages for categorical variables. Given the high inter-individual variability, most of the continuous data produced in this paper were non-normally distributed (assessed via Kolmogorov-Smirnov, Shapiro-Wilk tests, and visual inspection of Q-Q plots). Thus median (interquartile [Q] or [25%, 75%]) thus was used. When appropriate, parametric tests were computed, but for the most part, the non-parametric alternative had to be adopted. Mann-Whitney U test and Kruskal-Wallis test were used, respectively, to compare means of groups of variables skewly distributed. The frequencies of categorical variables were compared using Pearson χ2 or Fisher’s exact test, when appropriate. Bonferroni correction was used to correct multiple testing. Logistic regression and correlation analyses were performed. A *p* value less than 0.05 was considered statistically significant. Odds ratios (ORs) and 95% confidence interval (CI) were also calculated using SSPS 19.0 V software (SPSS Inc., Chicago, IL, USA).

## CONCLUSIONS

We present a new low-frequency risk variant, *ESR1* rs9340803, in both LOAD and MCI cases, which might possess etiological relation to AD along the whole disease continuum. This *ESR1* variation independently or synergistically with *APOE*, elevates the risk of cognitive damaging for cases in our study. We put forward that, for the first time, this variation on *ESR1*, which might have functional impact by modulating the cholesterol content in brain and thus promoting Aβ production, could be a causal factor among the complex genetic pathological basis of AD.

## SUPPLEMENTARY MATERIAL

Supplementary Tables and Figures

Supplementary Materials and Methods

## References

[1] Reitz C, Brayne C, Mayeux R. Epidemiology of Alzheimer disease. Nat Rev Neurol. 2011; 7:137–52. 10.1038/nrneurol.2011.2 21304480 PMC3339565

[2] Raj T, Shulman JM, Keenan BT, Chibnik LB, Evans DA, Bennett DA, Stranger BE, De Jager PL. Alzheimer disease susceptibility loci: evidence for a protein network under natural selection. Am J Hum Genet. 2012; 90:720–26. 10.1016/j.ajhg.2012.02.022 22482808 PMC3322230

[3] Apostolova LG, Risacher SL, Duran T, Stage EC, Goukasian N, West JD, Do TM, Grotts J, Wilhalme H, Nho K, Phillips M, Elashoff D, Saykin AJ, and Alzheimer’s Disease Neuroimaging Initiative. Associations of the Top 20 Alzheimer Disease risk variants with brain amyloidosis. JAMA Neurol. 2018; 75:328–41. 10.1001/jamaneurol.2017.4198 29340569 PMC5885860

[4] Karch CM, Goate AM. Alzheimer’s disease risk genes and mechanisms of disease pathogenesis. Biol Psychiatry. 2015; 77:43–51. 10.1016/j.biopsych.2014.05.006 24951455 PMC4234692

[5] Solomon A, Kivipelto M. Cholesterol-modifying strategies for Alzheimer’s disease. Expert Rev Neurother. 2009; 9:695–709. 10.1586/ern.09.25 19402779

[6] Puglielli L, Tanzi RE, Kovacs DM. Alzheimer’s disease: the cholesterol connection. Nat Neurosci. 2003; 6:345–51. 10.1038/nn0403-345 12658281

[7] Di Paolo G, Kim TW. Linking lipids to Alzheimer’s disease: cholesterol and beyond. Nat Rev Neurosci. 2011; 12:284–96. 10.1038/nrn3012 21448224 PMC3321383

[8] Xiong H, Callaghan D, Jones A, Walker DG, Lue LF, Beach TG, Sue LI, Woulfe J, Xu H, Stanimirovic DB, Zhang W. Cholesterol retention in Alzheimer’s brain is responsible for high beta- and gamma-secretase activities and Abeta production. Neurobiol Dis. 2008; 29:422–37. 10.1016/j.nbd.2007.10.005 18086530 PMC2720683

[9] Donkin JJ, Stukas S, Hirsch-Reinshagen V, Namjoshi D, Wilkinson A, May S, Chan J, Fan J, Collins J, Wellington CL. ATP-binding cassette transporter A1 mediates the beneficial effects of the liver X receptor agonist GW3965 on object recognition memory and amyloid burden in amyloid precursor protein/presenilin 1 mice. J Biol Chem. 2010; 285:34144–54. 10.1074/jbc.M110.108100 20739291 PMC2962513

[10] Lehmann DJ, Butler HT, Warden DR, Combrinck M, King E, Nicoll JA, Budge MM, de Jager CA, Hogervorst E, Esiri MM, Ragoussis J, Smith AD. Association of the androgen receptor CAG repeat polymorphism with Alzheimer’s disease in men. Neurosci Lett. 2003; 340:87–90. 10.1016/S0304-3940(03)00069-7 12668243

[11] Hoffmann JM, Partridge L. Nuclear hormone receptors: roles of xenobiotic detoxification and sterol homeostasis in healthy aging. Crit Rev Biochem Mol Biol. 2015; 50:380–92. 10.3109/10409238.2015.1067186 26383043

[12] Etgen AM. Estrogens and Alzheimer’s disease: is cholesterol a link? Endocrinology. 2008; 149:4253–55. 10.1210/en.2008-0861 18723873

[13] Piscopo P, Tosto G, Belli C, Talarico G, Galimberti D, Gasparini M, Canevelli M, Poleggi A, Crestini A, Albani D, Forloni G, Lucca U, Quadri P, et al. SORL1 gene is associated with the conversion from Mild Cognitive Impairment to Alzheimer’s Disease. J Alzheimers Dis. 2015; 46:771–76. 10.3233/JAD-141551 25881907

[14] Tan L, Wang HF, Tan MS, Tan CC, Zhu XC, Miao D, Yu WJ, Jiang T, Tan L, Yu JT, Weiner MW, Aisen P, Petersen R, et al, and Alzheimer’s Disease Neuroimaging Initiative. Effect of CLU genetic variants on cerebrospinal fluid and neuroimaging markers in healthy, mild cognitive impairment and Alzheimer’s disease cohorts. Sci Rep. 2016; 6:26027. 10.1038/srep26027 27229352 PMC4882617

[15] Leduc V, De Beaumont L, Théroux L, Dea D, Aisen P, Petersen RC, Dufour R, Poirier J, and Alzheimer’s Disease Neuroimaging Initiative. HMGCR is a genetic modifier for risk, age of onset and MCI conversion to Alzheimer’s disease in a three cohorts study. Mol Psychiatry. 2015; 20:867–73. 10.1038/mp.2014.81 25023145 PMC4318698

[16] Welander H, Frånberg J, Graff C, Sundström E, Winblad B, Tjernberg LO. Abeta43 is more frequent than Abeta40 in amyloid plaque cores from Alzheimer disease brains. J Neurochem. 2009; 110:697–706. 10.1111/j.1471-4159.2009.06170.x 19457079

[17] Rolstad S, Berg AI, Bjerke M, Blennow K, Johansson B, Zetterberg H, Wallin A. Amyloid-β_42_ is associated with cognitive impairment in healthy elderly and subjective cognitive impairment. J Alzheimers Dis. 2011; 26:135–42. 10.3233/JAD-2011-110038 21593572

[18] Bettcher BM, Ard MC, Reed BR, Benitez A, Simmons A, Larson EB, Sonnen JA, Montine TJ, Li G, Keene CD, Crane PK, Mungas D. Association between cholesterol exposure and neuropathological findings: the ACT Study. J Alzheimers Dis. 2017; 59:1307–15. 10.3233/JAD-161224 28731431 PMC5604311

[19] Cedazo-Mínguez A, Ismail MA, Mateos L. Plasma cholesterol and risk for late-onset Alzheimer’s disease. Expert Rev Neurother. 2011; 11:495–98. 10.1586/ern.11.36 21469922

[20] Saykin AJ, Shen L, Yao X, Kim S, Nho K, Risacher SL, Ramanan VK, Foroud TM, Faber KM, Sarwar N, Munsie LM, Hu X, Soares HD, et al, and Alzheimer’s Disease Neuroimaging Initiative. Genetic studies of quantitative MCI and AD phenotypes in ADNI: Progress, opportunities, and plans. Alzheimers Dement. 2015; 11:792–814. 10.1016/j.jalz.2015.05.009 26194313 PMC4510473

[21] Bae JB, Kim YJ, Han JW, Kim TH, Park JH, Lee SB, Lee JJ, Jeong HG, Kim JL, Jhoo JH, Yoon JC, Kim KW. Incidence of and risk factors for Alzheimer’s disease and mild cognitive impairment in Korean elderly. Dement Geriatr Cogn Disord. 2015; 39:105–15. 10.1159/000366555 25401488

[22] Marquer C, Devauges V, Cossec JC, Liot G, Lécart S, Saudou F, Duyckaerts C, Lévêque-Fort S, Potier MC. Local cholesterol increase triggers amyloid precursor protein-Bace1 clustering in lipid rafts and rapid endocytosis. FASEB J. 2011; 25:1295–305. 10.1096/fj.10-168633 21257714

[23] Peri A, Benvenuti S, Luciani P, Deledda C, Cellai I. Membrane cholesterol as a mediator of the neuroprotective effects of estrogens. Neuroscience. 2011; 191:107–17. 10.1016/j.neuroscience.2011.03.011 21396986

[24] Yadav RS, Tiwari NK. Lipid integration in neurodegeneration: an overview of Alzheimer’s disease. Mol Neurobiol. 2014; 50:168–76. 10.1007/s12035-014-8661-5 24590317

[25] Chen LH, Fan YH, Kao PY, Ho DT, Ha JC, Chu LW, Song YQ. Genetic polymorphisms in estrogen metabolic pathway associated with risks of Alzheimer’s Disease: evidence from a southern Chinese population. J Am Geriatr Soc. 2017; 65:332–39. 10.1111/jgs.14537 28102888

[26] Fernández-Martínez M, Elcoroaristizabal Martín X, Blanco Martín E, Galdos Alcelay L, Ugarriza Serrano I, Gómez Busto F, Alvarez-Álvarez M, Molano Salazar A, Bereincua Gandarias R, Inglés Borda S, Uterga Valiente JM, Indakoetxea Juanbeltz B, Gómez Beldarraín MÁ, et al. Oestrogen receptor polymorphisms are an associated risk factor for mild cognitive impairment and Alzheimer disease in women APOE varepsilon4 carriers: a case-control study. BMJ Open. 2013; 3:e003200. 10.1136/bmjopen-2013-003200 24052609 PMC3780298

[27] Elcoroaristizabal Martín X, Fernández Martínez M, Galdos Alcelay L, Molano Salazar A, Bereincua Gandarias R, Inglés Borda S, Gómez Busto F, Uterga Valiente JM, Indakoetxea Juanbeltz B, Gómez Beldarraín MA, de Pancorbo MM. Progression from amnesic mild cognitive impairment to Alzheimer’s disease: ESR1 and ESR2 polymorphisms and APOE gene. Dement Geriatr Cogn Disord. 2011; 32:332–41. 10.1159/000335541 22311091

[28] Peri A. Neuroprotective effects of estrogens: the role of cholesterol. J Endocrinol Invest. 2016; 39:11–18. 10.1007/s40618-015-0332-5 26084445

[29] Morinaga A, Ono K, Takasaki J, Ikeda T, Hirohata M, Yamada M. Effects of sex hormones on Alzheimer’s disease-associated β-amyloid oligomer formation in vitro. Exp Neurol. 2011; 228:298–302. 10.1016/j.expneurol.2011.01.011 21281631

[30] Kölsch H, Lütjohann D, Jessen F, Popp J, Hentschel F, Kelemen P, Friedrichs S, Maier TA, Heun R. RXRA gene variations influence Alzheimer’s disease risk and cholesterol metabolism. J Cell Mol Med. 2009; 13:589–98. 10.1111/j.1582-4934.2009.00383.x 19374686 PMC3822518

[31] Wang L, Hara K, Van Baaren JM, Price JC, Beecham GW, Gallins PJ, Whitehead PL, Wang G, Lu C, Slifer MA, Züchner S, Martin ER, Mash D, et al. Vitamin D receptor and Alzheimer’s disease: a genetic and functional study. Neurobiol Aging. 2012; 33:1844.e1–9. 10.1016/j.neurobiolaging.2011.12.038 22306846

[32] McKhann GM, Knopman DS, Chertkow H, Hyman BT, Jack CR Jr, Kawas CH, Klunk WE, Koroshetz WJ, Manly JJ, Mayeux R, Mohs RC, Morris JC, Rossor MN, et al. The diagnosis of dementia due to Alzheimer’s disease: recommendations from the National Institute on Aging-Alzheimer’s Association workgroups on diagnostic guidelines for Alzheimer’s disease. Alzheimers Dement. 2011; 7:263–69. 10.1016/j.jalz.2011.03.005 21514250 PMC3312024

[33] Weiner MW, Veitch DP, Aisen PS, Beckett LA, Cairns NJ, Cedarbaum J, Green RC, Harvey D, Jack CR, Jagust W, Luthman J, Morris JC, Petersen RC, et al, and Alzheimer’s Disease Neuroimaging Initiative. 2014 Update of the Alzheimer’s Disease Neuroimaging Initiative: A review of papers published since its inception. Alzheimers Dement. 2015; 11:e1–120. 10.1016/j.jalz.2014.11.001 26073027 PMC5469297

[34] Albert MS, DeKosky ST, Dickson D, Dubois B, Feldman HH, Fox NC, Gamst A, Holtzman DM, Jagust WJ, Petersen RC, Snyder PJ, Carrillo MC, Thies B, Phelps CH. The diagnosis of mild cognitive impairment due to Alzheimer’s disease: recommendations from the National Institute on Aging-Alzheimer’s Association workgroups on diagnostic guidelines for Alzheimer’s disease. Alzheimers Dement. 2011; 7:270–79. 10.1016/j.jalz.2011.03.008 21514249 PMC3312027

