# Supplementary material for: The etiological effect of a new low-frequency ESR1 variant on Mild Cognitive Impairment and Alzheimer’s Disease: a population-based study
Source: Aging (Albany NY). 2018 Sep 16;10(9):2316–37. doi: 10.18632/aging.101548 (PMC6188501; doi:10.18632/aging.101548)
Supplement: Supplementary Tables and Figures [file aging-10-101548-s001.pdf]

SUPPLEMENTARY MATERIALS

Table S1. Baseline characteristics of participants in the present study.

	AD			MCI			CN		
	Total	F	M	Total	F	M	Total	F	M
No.(%)	854	451 (52.8%)	403 (47.2%)	1059	678 (57.4%)	381 (42.6%)	1254	689 (54.9%)	565 (45.1%)
Age(y)	77.5(14)	79(13)	81(14)	73(12)	69(12)	72(10)	65(10)	67(9)	68(11)
E+	37	15	22	43	28	15	17	8	9
A+	257	133	124	208	75	133	212	118	94

No.:number; F:female; M:male; E+: *ESR1* rs9340803 G allele carrier； A+: *APOEε4* carrier.

Discovery of NR gene variants using targeted NGS

The sequencing data yielded, on average, 125.5 Mb of 100 bp paired-end sequence reads per individual, representing an average coverage depth of approximately 126X. Approximately 85.3% of the sequence reads were mapped to unique regions of the human genome (Build 37.5, hg19; BWA software). The Samtools software called out, on average, 134 single nucleotide variations (SNVs) per individual compared to the reference genome.

In all, we identified 1690 SNVs. Among those, 329 SNVs were consistent with those in the public SNP database and 1361 SNVs were previously unknown. 100 SNVs resided within putative promoter regions of the 13 genes and 1564 SNPs were located in the introns, all known exons, untranslated regions, or splice sites. A total of 26 SNPs were within non-coding RNA intronic and exonic regions (Table S2).

Table S2. The summary of SNVs and indels discovered in targeted sequencing stage.

Variant type	No. of known SNVs	No. of novel SNVs	Indels
Putative Promoter	17	83	13
Intron	172	1194	131
3’UTR	95	36	44
5’UTR	9	3	1
Exon	36	18	12
NcRNA_Intron	0	15	1
NcRNA_Exon	0	11	1
Splice site	0	1	9
Total	329	1361	213

Table S3. Allele and genotype frequencies of *ESR1* rs9340803.

	CN	case	*p*(G/A)	OR	95%CI	CN	case	*p*(GA/AA)	OR	95%CI
a	MAF<0.01	4/142	<0.001	7.4	2.48~10.84	MAF<0.01	4/69	<0.001	7.58	2.51~13.06
C1	4/386	15/389	0.01	3.72	1.22~5.53	4/191	15/187	0.01	3.83	1.25~6.73
C2	13/2105	18/1140	0.008	2.56	1.25~3.13	13/1046	18/561	0.008	2.58	1.26~3.43
C3	17/2511	37/1671	<0.001	3.27	1.84~3.89	17/1237	37/817	<0.001	3.30	1.84~4.22
b	17/2511	43/2075	<0.001	3.15	1.86~3.64	17/1237	43/1016	<0.001	3.18	1.87~3.89
C4	17/2511	80/3746	<0.001	3.06	1.74~3.61	17/1237	80/1833	<0.001	3.08	1.75~3.89

C1: sample group1(200 LOAD case vs. 200 controls); C2: sample group 2(581 LOAD cases vs. 1054 cases); C3: combined sample group (854 LOAD cases vs. 1254 controls); a: data from the 1000 GENOME; b: 1059 MCI cases vs. 1254 controls; C4: 1913 CI cases vs. 1254 controls.

Table S4. Stratified analyses of rs9340803 distribution.

			E+	E-		*p*	pa		
All		AD	37	817	854		0.059		
		MCI	43	1016	1059	<0.001		<0.001	
		CN	17	1237	1254				<0.001
		Sum	97	3070	3167				
Gender		AD	15	436	451				
	F	MCI	8	681	689	0.003	0.490		
		CN	28	650	678			0.001	
		Sum	51	1767	1818				0.011
		AD	22	381	403				
		MCI	9	556	565		0.315		
	M	CN	15	366	381	0.004		0.025	0.001
		Sum	46	1303	1349				
Region		AD	15	551	566		0.232		
	S	MCI	35	864	899	0.002		<0.001	
		CN	8	742	750				0.03
		Sum	58	2157	2215				
		AD	22	266	288		0.268		
	N	MCI	8	152	160	<0.001		<0.001	
		CN	9	495	504				<0.001
		Sum	39	913	952				
APOE		AD	12	245	257		0.140		
	A+	MCI	7	201	208	0.141		0.190	
		CN	3	209	212				0.046
		Sum	22	655	677				
		AD	25	572	597		0.008		
	A-	MCI	36	815	851	<0.001		0.625	
		CN	14	1028	1042				0.554
		Sum	75	2415	2490				
AG		AD	8	196	204		0.056		
	<70y	MCI	13	365	378	0.025		0.022	
		CN	13	885	898				0.020
		Sum	34	1446	1480				
		AD	29	621	650		0.010		
	≥70y	MCI	30	651	681	0.014		0.005	
		CN	4	352	356				0.005
		Sum	63	1624	1687				

E-: *ESR1* rs9340803 A allele carrier; N: northern; S: southern; A-: non- *APOE*ε4 carrier; AG: age group.

Table S5. Comparisons between CI cases and CN on rs9340803 G allele.

	E+	E-	χ2	*p*	OR(95%CI)
CI	80	1833	20.38	<0.001	3.18(1.87-3.85)
CN	17	1237			
	A+	A-			
CI	465	1448	24.69	<0.001	1.58(1.32-1.95)
CN	212	1042			
	E+ A+	E+A-			
CI	19	61	0.714	0.398	-
CN	3	14			
	A-/E+	A-/E-			
CI	61	1387	17.08	<0.001	3.23(1.80~4.05)
CN	14	1028			
	E-/A+	E-/A-			
CI	446	1387	24.33	<0.001	1.58(1.32-1.96)
CN	209	1028			
	E+/A+	E-/A-			
CI	19	1387	7.48	0.006	4.69(1.39-5.89)
CN	3	1028			

CI: cognitive impairment; MT: G allele carrier; WT:A allele carrier

Table S6. Logistic analysis of CI with gene variants, gender and age.

	B	S.E,	Wals	df	Sig.	Exp (B)	95% CI of EXP(B)	
							lower	upper
apoe4	0.382	0.101	14.426	1	0.000	1.466	1.203	1.785
gender	-0.362	0.082	19.587	1	0.000	0.696	0.593	0.817
age	1.777	0.081	475.74	1	0.000	5.911	5.038	6.934
esr1mut	1.137	0.288	15.603	1	0.000	3.118	1.774	5.483

Table S7. Stratified analysis between CI cases and CNs on rs9340803 G allele.

		E+	E-	*p*	OR(95%CI)
F	CI	43	1086	<0.001	3.37(1.58~4.40)
	CN	8	681		
M	CI	37	757	0.002	3.06(1.46~4.16)
	CN	9	556		
<70	CI	21	561	0.007	2.55(1.27~3.42)
	CN	13	885		
≥70	CI	59	1272	0.003	4.08(1.47~5.60)
	CN	4	352		

Table S8. Non-*APOE*ε4-stratified comparisons between CI cases and CNs on rs9340803 G allele.

A-		E+	E-	*p*	OR(95%CI)
F	CI	9	309	0.048	2.74(0.97~4.03)
	CN	6	565		
M	CI	16	263	0.002	3.52(1.49~5.36)
	CN	8	463		

Gene-gene & gene-environment interaction

Compared with CNs, ESR1 rs9340803 G allele and APOE4 synergistically elevating the effect size to 4.69-fold(1.39-5.89) among AD or MCI patients. Given the preliminary results and the fact that aging was the most prominent risk factor, we're promoted to ask whether the identified new low-frequency ESR1 mutation, APOE4 together with aging may collectively contribute to the development of AD. Therefore, gene-gene interaction and gene-circumstance(aging) interaction were explored using GMDR software(https://sourceforge.net/projects/gmdr/). It turned out that one three locus-aging model, ESR1 (rs9340803)-APOE (rs429358, rs7412)-aging, had a maximum testing accuracy of 71.22% and a maximum cross-validation consistency (100/100) that was significant at $p<0.0001$ level. In the three-locus(rs1387923–rs2769605–rs6265) model, the ORs for the three high-risk genotype combinations (AG)-(TT)-(CC), (AA)-(CC)-(CC), and (AA)-(TC)-(CC) were 2.4(95% CI: 1.2-3.2), 4.7 (95% CI: 1.7-6.3) and 1.3 (95% CI: 1.1-1.7), respectively. Traditional statistical method was utilized in parallel, in order to further validate the risk-associated genotype and haplotype additionally, turne out that: 1) AG-TT-CC genotype occupied the potential of increasing of disease risk to 2.4(1.2-3.2)-fold in specific population, while to 6.30(0.85-10.14)-fold in individuals 70 years and more at age, while the other two genotypes didn't reach the statistically significance($p=0.821$, $p=0.051$, respectively); 2) the corresponding risk added up to 2.46 (1.18-3.29)-fold in the elderly with G-T-C haplotype, and to 6.54(0.88-10.52) if aged 70 or older. Besides, multinomial logistic regression analysis was also conducted, of which the result indicated that ESR1 rs9340803, APOE and aging would contribute in joint to the risk of cognitive devastation associated with AD.

Table S9. Genotype and haplotype analysis of rs9340803, rs429358 and rs7412.

Genotype	CI	CN	χ2	*p*	OR(95%CI)
AG-TT-CC	39	9	5.81	0.02	2.4(1.2-3.2)
AA-CC-CC	34	4	10.26	0.001	4.7 (1.7-6.3)
AA-TC-CC	288	121	5.20	0.02	1.3 (1.1-1.7)
Age≥70	CI	CN	χ2	*p*	OR(95%CI)
AG-TT-CC	27	1	4.25	0.04	6.30(0.85-10.14)
Haplotype	CI	CN	χ2	*p*	OR(95%CI)
A-C-C	322	125	9.03	0.002	1.42 (1.13-1.88)
G-T-C	40	9	6.20	0.01	2.46 (1.18-3.29)
G-C-C	4	2	0.01	0.91	1.11
A-T-T	215	118	0.00	0.95	1.01
A-C-T	23	13	0.00	0.95	0.98
G-T-T	7	4	0.00	0.96	0.97
G-C-T	2	1	0.01	0.93	1.11
Age≥70	CI	CN	χ2	*p*	OR(95%CI)
A-C-C	220	38	2.33	0.13	1.35(0.92-2.12)
G-T-C	28	1	4.47	0.03	6.54(0.88-10.52)

Table S10. Stratified analyses on MMSE.

	Median(25%, 75%)		*p*
E+/-	14(10, 20)	15(9, 21)	0.874
A+/-	16(9, 22)	15(9, 20)	0.178
A-E+/E-	16.5(10.5, 20)	15(9, 20)	0.423
F/M	14(9, 20)	16(10, 21)	0.103
≥70/<70	14(9, 20)	18(13, 22)	<0.001

Table S11. Analyses on serum Aβ-oligomer concentrations ((pmol/L)).

Aβ	AD/MCI/CN			*p*	CI/CN		*p*	F/M		*p*	≥70/<70		*p*
40	39.69 (21.98, 53.50)	29.63 (15.47, 47.92)	16.52 (4.84, 44.64)	<0.001	32.67 (16.52, 49.81)	16.52 (4.84, 44.64)	<0.001	30.49 (12.02, 47.87)	29.92 (12.20, 49.20)	0.693	34.06 (15.60, 52.65)	25.11 （8.07, 42.62)	<0.01
42	3.68 (2.17, 5.72)	2.74 (1.22, 4.60)	2.25 (1.08, 4.15)	<0.001	3.06 (1.41, 4.87)	2.25 (1.08, 4.15)	<0.001	2.90 (1.41, 4.69)	2.72 (1.16, 4.70)	0.212	3.22 (1.44, 5.04)	2.42 (1.16, 4.28)	>0.05
42/40	0.103 (0.078, 0.135)	0.097 (0.073, 0.117)	0.130 (0.081, 0.254)	<0.001	0.10 (0.07, 0.12)	0.13 (0.08, 0.25)	<0.001	0.103 (0.078, 0.140)	0.098 (0.070, 0.134)	0.003	0.098 (0.073, 0.127)	0.105 (0.079, 0.159)	>0.05

Table S12. Stratified analyses on serum Aβ-oligomers ((pmol/L)).

	E+/-		*p*	A+/-		*p*	A-E+/-		*p*
40	35.56 (14.84, 57.44)	30.14 (11.98, 48.21)	0.045	33.05 (15.25, 52.09)	29.69 (11.48, 47.80)	0.01	30.75 (10.59, 56.93)	29.65 (11.35, 47.42)	0.163
42	3.76 (1.43, 5.82)	2.80 (1.28, 4.67)	0.124	2.88 (1.33, 4.91)	2.80 (1.29, 4.63)	0.468	3.14 (1.43, 5.79)	2.78 (1.28, 4.59)	0.274
42/40	0.098 (0.080, 0.125)	0.102 (0.075, 0.138)	0.563	0.099 (0.073, 0.124)	0.103 (0.076, 0.141)	0.026	0.099 (0.077, 0.122)	0.103 (0.076, 0.142)	0.457

Table S13. Four major items of blood lipids (mmol/L).

	AD/MCI/CN			*p*	CI/CN		*p*	F/M		*p*	≥70/<70		*p*
Tch	4.34 (3.12, 5.30)	4.82 (4.19, 5.44)	5.09 (4.59, 5.72)	<0.001	4.59 (3.75, 5.37)	5.09 (4.59, 5.72)	<0.001	4.98 (4.16, 5.68)	4.56 (3.69, 5.25)	<0.001	4.46 (3.55, 5.22)	5.20 (4.64, 5.75)	<0.001
TG	1.33 (0.88, 2.83)	1.36 (0.97, 1.76)	1.16 (0.91, 1.74)	0.062	1.35 (0.9, 2.01)	1.16 (0.91, 1.74)	0.027	1.22 (0.86, 1.97)	2.72 (1.16, 4.70)	0.243	1.28 (0.9, 1.94)	1.31 (0.91, 1.84)	0.704
HDL	1.33 (1.11, 1.56)	1.25 (1.09, 1.47)	1.3 (1.11, 1.62)	0.051	1.29 (1.1, 1.51)	1.3 (1.11, 1.62)	0.103	1.34 (1.15, 1.62)	0.098 (0.070, 0.134)	<0.001	1.27 (1.1, 1.5)	1.31 (1.14, 1.61)	0.037
LDL	2.74 (2.09, 3.33)	2.71 (2.24, 3.22)	2.84 (2.46, 3.34)	0.324	2.73 (2.18, 3.3)	2.84 (2.46, 3.34)	0.144	2.85 (2.32, 3.41)	2.63 (2.09, 3.24)	0.008	2.62 (2.06, 3.15)	2.97 (2.52, 3.53)	<0.001

Tch: total cholesterol; TG: triglyceride; HDL-c: high density lipoprotein cholesterol; LDL-c: low density lipoprotein cholesterol.

Table S14. Comparisons on blood lipids (mmol/L).

	E+/-		*p*	A+/-		p	A-E+/-		*p*
Tch	3.96 (1.39, 5.42)	4.79 (3.96, 5.48)	0.045	4.65 (3.75, 5.39)	4.81 (3.96, 5.51)	0.294	4.19 (1.47, 5.56)	4.81 (3.96, 5.51)	0.173
TG	1.58 (0.92, 4.33)	1.28 (0.90, 1.92)	0.315	1.25 (0.94, 2.02)	1.29 (0.89, 1.91)	0.702	1.71 (1.07, 4.27)	1.29 (0.89, 1.91)	0.189
HDL	1.35 (1.11, 1.57)	1.29 (1.10, 1.54)	0.729	1.32 (1.10, 1.58)	1.29 (1.1, 1.53)	0.663	1.34 (1.09, 1.56)	1.29 (1.1, 1.53)	0.909
LDL	2.47 (1.69, 3.52)	2.76 (2.23, 3.31)	0.293	2.71 (2.20, 3.37)	2.78 (2.22, 3.3)	0.792	2.75 (1.46, 3.56)	2.78 (2.22, 3.3)	0.626

a: AD/MCI/CN, $p<0.0167$ using Kruskal-Wallis Test; b: CI/CN, $p<0.05$ using Mann-Whitney Test.

Functional prediction for the LOAD-associated variant

We explored the role of *ERS1* rs9340803 G allele in the cytological level preliminarily. Rs9340803A /G was located in the intron 4 of *ERS1* gene, close to the 3' receptor site splicing region of exon 4. MutationTaster, Human Splicing Finder and SFmap were used to assess the potential impact of rs9340803 G variant on *ERS1* alternative splicing, and this variant was pridicted to damage the regulation of intrinsic splicing of precursor *ERS1*mRNA. In addition, SFmap predicted that the G allele variant would destroy the binding site for the hnRNP H1, and Human Splicing Finder predicted it to generate a binding site for hnRNP A1, which is known to promote exon exclusion and induced abnormal exon skipping.

A

gDNA Position	Linked hnRNP protein (threshold values)	Mutant Motif (value 0-100)	Variation
g.186142	hnRNP A1(65.476)	gagcGg (70.48)	New site

B

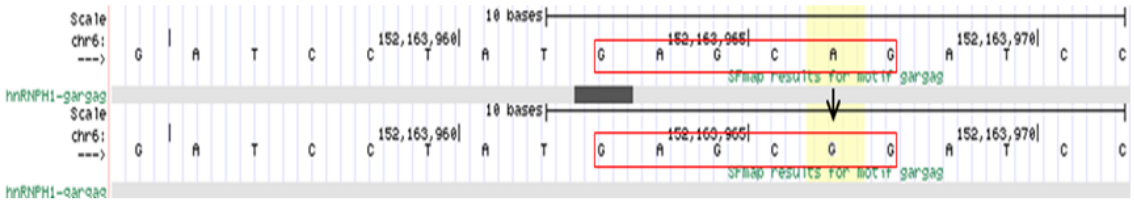

Figure S1. Functional damaging prediction for rs9340803 A/G variant. (**A**). The potential effect of the rs9340803A/G on *ESR1* alternative splicing predicted by HSF. The binding site for hnRNP A1is predicted to generate. (**B**). The potential effect of the rs9340803A/G on *ESR1* alternative splicing predicted using Sfmap. This variant is precited to cause the binding site of hnRNP H1 broken which targets the exonic splicing regulatory sequence(gagcag). Green bars present ESR1 binding sites of hnRNPH1. The arrows show the rs9340803 A to G change.
