# Supplementary material for: The etiological effect of a new low-frequency ESR1 variant on Mild Cognitive Impairment and Alzheimer’s Disease: a population-based study
Source: Aging (Albany NY). 2018 Sep 16;10(9):2316–37. doi: 10.18632/aging.101548 (PMC6188501; doi:10.18632/aging.101548)
Supplement: Supplementary Materials and Methods [file aging-10-101548-s002.pdf]

SUPPLEMENTARY MATERIALS AND METHODS

Participants

A total of 5635 Han Chinese participants were enrolled to our study from November 2012 through December 2017. Samples were collected from the Chinese PLA No. 253 Hospital in Inner Mongolia, Beijing Hospital, Beijing geriatric hospital, Chinese PLA General Hospital, Affiliated Rehabilitation Hospital of the National Rehabilitation Aids Research Center, China-Japan Friendship Hospital in Beijing, Chinese PLA No. 401 Hospital in Shangdong Province in North China, Affiliated Foshan Hospital of Sun Yat-sen University in Guangdong Province, Jiangbin Hospital of Guangxi Zhuang Autonomous Region in Guangxi Province in South China, and the Chinese Center For Disease Control And Prevention(CDC) covering Beijing, Shanghai, Hubei Province, Sichuan Province, Yunnan Province, Guangxi Province, with great help of assigned senior cinlinians.

The procedures of the present study were approved by the ethical review boards at all involved study centers and written informed consent was obtained from each subject or proxy.

Patients with other type of dementia such as frontotemporal dementia (FTD), dementia with Lewy bodies (DLB), Parkinson's dementia (PD) and vascular dementia (VD) or dementia caused by other factors such as multiple sclerosis will be excluded with cautious differential diagnoses. People with a history of alcoholism or drug abuse or for whom structural neuroimaging didn't support or ruled out a diagnosis of AD were also excluded. Criteria for undiagnosed AD patients included 1) an MMSE score lower than 27; 2) exclusion of other likely types of dementia; and 3) lacking of imaging examination at the time of recruitment.

Among the overall enrollees, 2468 cases were excluded, 43 AD cases, 101 MCI cases and 2324 CNs, to be specific, with respect to the corresponding conditions likeI) failure on *APOE* or *ESR1* genotyping; II) demographic information incomplete; III) age<50 years old. Thus, 3167 individuals were investigated finally.

Selection of 12 candidate NR genes and *APOE*

Twelve candidate cholesterol regulating gene-related-nuclear receptor (NR) genes (VDR, THRA, ESR1, ESR2, LXRB, PPARA, PPARB, PPARG, AR, GR, RXRA and RXRB) were selected for next-generation sequencing basing on previous meta-analysis and bioinformatic pathway analysis, among which I) all are involved in the cholesterol metabolism; II) two genes, LXRB and ESR1, are associated with up-regulation of APOE expression; and III) four genes, RXRA, VDR, ESR1 and AR, contain common LOAD-associated polymorphisms. Additionally, we incorporated APOE into this study.

Targeted sequencing

An approximately 150 kb genome region across the 12 NR genes and APOE were sequenced using pools of PCR productions from 73 Chinese LOAD patients. In brief, we amplified the putative promoter regions (3 kb upstream of the transcriptional start sites), all known exons, untranslated regions and the 200 bp intronic sequence flanking exons for detection of variation which affects alternative splicing of genes using PCR technique. Purified amplicons subsequently were used for constructing fragment libraries with Truseq DNA Sample Preparation Kit (Illumina San Diego, California, USA). Bar-coded fragment of sequencing libraries were added using a paired-end DNA sample preparation kit (Illumina, California, USA) and Illumina multiplexing adaptor (Illumina) according to the manufacturer's instructions. The quality control of libraries were tested utilizing real-time PCR with the LightCycler480IIsystem (Roche Madison, WI, USA). The 73 pooled libraries were then used for parallel sequencing with the utilization of a Hiseq 2000 sequencer (Illumina San Diego, California, USA). Sequencing data were aligned to the genomic reference (GRch37.5) using BWA software. Single nucleotide variants (SNVs) and small deletion and insertion (Indel) variants were called using SAMtools 1.31 and GATK 2.6, respectively and annotated by comparing with the dbSNP, HapMap and 1000 Genomes databases.

To discover new, low-frequency or rare variants that are associated with LOAD, we applied several rigorous analysis steps. We re-aligned the BWA-aligned reads using the Sequence Alignment/Map (SAM) tools 1.31 and the Genome Analysis Toolkit (GATK) 2.6. Potential SNVs and Indels were called using SAMtools. In this process, several heuristic rules were applied: (i) all samples should be covered sufficiently ($\geq 10\times$) at the genomic position being compared; (ii) the average base quality for a given genomic position should be at least 15 in all 73 pooled samples; (iii) The variants should be supported by at least 10% of the total reads; (iv) Each variant should be supported by at least five reads. To further reduce the false positive calls, SNVs and Indels called using the SAMtools were re-called with GATK software package in the 73 pooled samples. We discarded

variants that fulfill any one of the following filtering criteria: (i) variants with phred-like scaled consensus scores or SNP qualities < 20; (ii) variants with mapping qualities < 30; (iii) variants with more than 10% of the simulated variant-containing reads that could not be uniquely mapped to the reference genome. Those SNVs that were accorded with the filtering criteria above and were commonly called by SAMtools and GATK were kept for association tests.

SNVs selection

We selected low-frequency and rare variants for subsequent association tests because: I) variants located in repeat sequences, including short tandem repeats (STR) or single nucleotide repeat expansions, may be falsely called, and thus were discarded; II) Given that the potential disease-associated variants are likely to be those variants that are low frequency (1% ≤ MAF <5%) and rare (MAF <1%) in the population, but they are frequently carried in patients (MAF ≥5%), leading to variants with MAF <5% in population (referred to the 1000 Genomes database), but with MAF ≥5% in the 73 LOAD samples kept; III) To further reduce the false positive variants, those low-frequency or rare variants that were called out by both SAMtools and GATK software package were kept; IV) For the pooled samples, it was impossible to effectively distinguish low-frequency or rare Indel variants from sequencing errors, thus only SNVs in this study were analyzed.

Genotyping of candidate SNVs in small scale cohort

After candidates were validated, we genotyped 8 low-frequency or rare SNVs and APOE rs429358 using the MassARRAY Compact system (Sequenom, San Diego, CA) and the High-Resolution Melting (HRM) method in 200 LOAD cases and 200 controls. Briefly, PCR amplification of Chr12g.48272978 C>A, rs138110733, Chr19g.45406107 A>G, rs429358 and rs9340803 were carried out with 10ng DNA and SNP specific primers, followed by a base extension reaction using iPLEX Gold chemistry (Sequenom, San Diego, CA). The final base extension products were subsequently treated and spotted on a 384-pad SpectroCHIP (Sequenom) using a MassARRAY Samsung Nanodispenser (Sequenom, San Diego, CA). MassARRAY Analyzer Compact MALDI-TOF MS (Sequenom, San Diego, CA) was used for data acquisition from the SpectroCHIP. Resultant genotypes were called using MassARRAY TYPER V4.0 software. Meanwhile, rs377476609, rs9658164 and rs7038025 were genotyped using HRM. Rs429358 and rs9340803 were genotyped utilizing Sanger sequencing. Genotyping primers for the MassARRAY and HRM were claimable.

Quality control of genotyping

For those variants (VDR Chr12g.48272978, RXRA rs138110733, TOMM40 Chr19g.45406107 A>G, APOE rs429358 and ESR1 rs9340803) genotyped by MassARRAY, re-genotyping were carried out in 30% subjects, which were randomly sampled from subjects using MassARRAY platform. For ESR1 rs377476609, PPARD rs9658164 and RXRA rs7038025 genotyped by HRM, all subjects who carried the minor alleles, together with 10% cases and 10% controls who carried the major alleles were re-genotyped using Sanger sequencing.
